# Supplementary material for: A mechanosensitive caveolae–invadosome interplay drives matrix remodelling for cancer cell invasion
Source: Nat Cell Biol. 2023 Oct 30;25(12):1787–803. doi: 10.1038/s41556-023-01272-z (PMC10709148; doi:10.1038/s41556-023-01272-z)
Supplement: Supplementary file 1 — Reporting Summary [file 41556_2023_1272_MOESM1_ESM.pdf]

Corresponding author(s): Philippe Chavrier
Christophe Lamaze
Pedro Monteiro

Last updated by author(s): Sep 13, 2023

Reporting Summary

Nature Portfolio wishes to improve the reproducibility of the work that we publish. This form provides structure for consistency and transparency in reporting. For further information on Nature Portfolio policies, see our Editorial Policies and the Editorial Policy Checklist.

Statistics

For all statistical analyses, confirm that the following items are present in the figure legend, table legend, main text, or Methods section.

n/a	Confirmed	
☐	☒	The exact sample size (*n*) for each experimental group/condition, given as a discrete number and unit of measurement
☐	☒	A statement on whether measurements were taken from distinct samples or whether the same sample was measured repeatedly
☐	☒	The statistical test(s) used AND whether they are one- or two-sided<br>*Only common tests should be described solely by name; describe more complex techniques in the Methods section.*
☒	☐	A description of all covariates tested
☐	☒	A description of any assumptions or corrections, such as tests of normality and adjustment for multiple comparisons
☐	☒	A full description of the statistical parameters including central tendency (e.g. means) or other basic estimates (e.g. regression coefficient) AND variation (e.g. standard deviation) or associated estimates of uncertainty (e.g. confidence intervals)
☐	☒	For null hypothesis testing, the test statistic (e.g. *F*, *t*, *r*) with confidence intervals, effect sizes, degrees of freedom and *P* value noted<br>*Give P values as exact values whenever suitable.*
☒	☐	For Bayesian analysis, information on the choice of priors and Markov chain Monte Carlo settings
☒	☐	For hierarchical and complex designs, identification of the appropriate level for tests and full reporting of outcomes
☐	☒	Estimates of effect sizes (e.g. Cohen's *d*, Pearson's *r*), indicating how they were calculated

Our web collection on statistics for biologists contains articles on many of the points above.

Software and code

Policy information about availability of computer code

Data collection	NIS Elements (version 5.42.01) and Metamorph (version 7.8.0.0) softwares were used for data collection.<br>BioRad ChemiDoc MP Imaging System (version 6.1.0.07) was used for western blot analysis.
Data analysis	Fiji software (version 1.54) was used for data analysis.<br>GraphPad Prism (versions 8.0, 9.0 and 10.0) software was used for statistic calculations and data representation.

For manuscripts utilizing custom algorithms or software that are central to the research but not yet described in published literature, software must be made available to editors and reviewers. We strongly encourage code deposition in a community repository (e.g. GitHub). See the Nature Portfolio guidelines for submitting code & software for further information.

Data

Policy information about availability of data

All manuscripts must include a data availability statement. This statement should provide the following information, where applicable:

- Accession codes, unique identifiers, or web links for publicly available datasets
- A description of any restrictions on data availability
- For clinical datasets or third party data, please ensure that the statement adheres to our policy

The authors declare that the data supporting the findings of this study are available within the main and supplementary figures of this paper. All other data

supporting the findings are available on request.

Human research participants

Policy information about studies involving human research participants and Sex and Gender in Research.

Reporting on sex and gender	N/A
Population characteristics	N/A
Recruitment	N/A
Ethics oversight	N/A

Note that full information on the approval of the study protocol must also be provided in the manuscript.

Field-specific reporting

Please select the one below that is the best fit for your research. If you are not sure, read the appropriate sections before making your selection.

☒ Life sciences ☐ Behavioural & social sciences ☐ Ecological, evolutionary & environmental sciences

For a reference copy of the document with all sections, see nature.com/documents/nr-reporting-summary-flat.pdf

Life sciences study design

All studies must disclose on these points even when the disclosure is negative.

Sample size	No pre-determined sample size was established. The number of experiments and sample size are indicated in Source data files. Sample size was determined based on previous studies in the field (Infante et al. Nat Commun 2018; Ferrari et al. Nat Commun 2019; Zagryazhskaya-Masson et al. JCB, 2020)
Data exclusions	No data were excluded.
Replication	All the experimental findings were reliably reproduced as validated by at least three independent experiments.
Randomization	This does not apply to the set of experiments presented in this paper where two distinct groups were generally compared and the order of analysis does not influence the experimental outcomes.
Blinding	The investigators were not blinded during data collection or analysis as the method of quantification over multiple replicates (for all experiments) and individual cells ensures unbiased processing. Additionally, blinding was not possible as the same researcher produced and analysed the data.

Reporting for specific materials, systems and methods

We require information from authors about some types of materials, experimental systems and methods used in many studies. Here, indicate whether each material, system or method listed is relevant to your study. If you are not sure if a list item applies to your research, read the appropriate section before selecting a response.

Materials & experimental systems

n/a	Involved in the study	
☐	☒	Antibodies
☐	☒	Eukaryotic cell lines
☒	☐	Palaeontology and archaeology
☒	☐	Animals and other organisms
☒	☐	Clinical data
☒	☐	Dual use research of concern

Methods

n/a	Involved in the study	
☒	☐	ChIP-seq
☒	☐	Flow cytometry
☒	☐	MRI-based neuroimaging

Antibodies

Antibodies used	Exhaustive description of antibodies used in this study are listed in Methods section and Extended Data Table 2.

Validation

All commercially available primary antibodies were validated for the use in westernblotting or immunofluorescence using siRNA approach.

Anti-TKS5, Cell Signaling Technology (#16619), validated for Western Blot and immunofluorescence analyses, relevant citations can be found on the manufacturer's website (https://www.cellsignal.com/products/primary-antibodies/tks5-antibody/16619).

Anti-MT1-MMP, Millipore (#3328), validated for Western Blot and immunofluorescence analyses, relevant citations can be found on the manufacturer's website (https://www.merckmillipore.com/FR/fr/product/Ms-X-MMP-14-Antibody-clone-LEM-2-15.8-MT1-MMP,MM_NF-MAB3328-25UG?ReferrerURL=https%3A%2F%2Fwww.google.com%2F).

Anti-Caveolin-1/Cav1, Cell Signaling Technology (#3238), validated for Western Blot and immunofluorescence analysis, relevant citations can be found on the manufacturer's website (https://www.cellsignal.com/products/primary-antibodies/caveolin-1-antibody/3238).

Anti-Cavin1, Abcam (#ab48824), validated for Western Blot and immunofluorescence analysis, relevant citations can be found on the manbetafacturer's website (https://www.abcam.com/products/primary-antibodies/ptrf-antibody-ab48824.html).

Anti-N-WASP, Cell Signaling Technology (#4848), validated for Western Blot analysis, relevant citations can be found on the manufacturer's website (https://www.cellsignal.com/products/primary-antibodies/n-wasp-30d10-rabbit-mab/4848).

Anti-beta1 integrin, BioLegend (#303036), validated for Western Blot and immunofluorescence analyses, relevant citations can be found on the manufacturer's website (https://www.biolegend.com/nl-nl/products/ultra-leaf-purified-anti-human-cd29-antibody-19166?GroupID=BLG10310).

Anti-beta3 integrin, Cell Signaling Technology (#13166), validated for Western Blot analysis, relevant citations can be found on the manufacturer's website (https://www.cellsignal.com/products/primary-antibodies/integrin-b3-d7x3p-xp-rabbit-mab/13166).

Anti-EHD2, Santa Cruz Biotechnology (#sc-100724), validated for Western Blot analysis, relevant citations can be found on the manufacturer's website (https://www.scbt.com/fr/p/ehd2-antibody-l-05).

Anti-Cortactin, Merck Millipore (#05-180), validated for Western Blot and immufluorescence analyses, relevant citations can be found on the manufacturer's website (https://www.merckmillipore.com/FR/fr/product/Anti-Cortactin-p80-85-Antibody-clone-4F11,MM_NF-05-180-I-100UL).

Anti-Col1-3/4C, ImmunoGlobe GmbH (#0217-050), validated for immufluorescence analysis, relevant citations can be found on the manufacturer's website (https://www.immunoglobe.com/antibodies/items/collagen_cleavage_site.html).

GAPDH, Santa Cruz Biotechnology (#sc-25778), validated for Western Blot analysis, relevant citations can be found on the manufacturer's website (https://www.scbt.com/fr/p/gapdh-antibody-fl-335).

Anti-CD63, BD Biosciences (#556019), validated for Western Blot and immunofluorescence analyses, relevant citations can be found on the manufacturer's website (https://www.bdbiosciences.com/en-at/products/reagents/flow-cytometry-reagents/research-reagents/single-color-antibodies-ruo/purified-mouse-anti-human-cd63.556019).

Anti-Rab5, Cell Signaling Technology (#3547), validated for Western Blot and immunofluorescence analyses, relevant citations can be found on the manufacturer's website (https://www.cellsignal.com/products/primary-antibodies/rab5-c8b1-rabbit-mab/3547).

Anti-Rab7, Cell Signaling Technology (#9367), validated for Western Blot and immunofluorescence analyses, relevant citations can be found on the manufacturer's website (https://www.cellsignal.com/products/primary-antibodies/rab7-d95f2-xp-rabbit-mab/9367).

Anti-GFP, Abcam (#ab13970), validated for Western Blot and immunofluorescence analyses, relevant citations can be found on the manufacturer's website. (https://www.abcam.com/products/primary-antibodies/gfp-antibody-ab13970.html).

Anti IgG-rabbit-A488, Molecular Probes (#A11034), validated for immunofluorescence analysis, relevant citations can be found on the manufacturer's website (https://www.thermofisher.com/antibody/product/Goat-anti-Rabbit-IgG-H-L-Highly-Cross-Adsorbed-Secondary-Antibody-Polyclonal/A-11034).

Anti-IgG-rabbit-Cy3, Jackson ImmunoResearch (#711-165-152), validated for immunofluorescence analysis, relevant citations can be found on the manufacturer's website (https://www.jacksonimmuno.com/catalog/products/711-165-152).

Anti-IgG-mouse-A488, Molecular Probes (#A21202), validated for immunofluorescence analysis, relevant citations can be found on the manufacturer's (https://www.thermofisher.com/antibody/product/Donkey-anti-Mouse-IgG-H-L-Highly-Cross-Adsorbed-Secondary-Antibody-Polyclonal/A-21202).

Anti-IgG-mouse-Cy3, Jackson ImmunoResearch (#711-165-151), validated for immunofluorescence analysis, relevant citations can be found on the manufacturer's website (https://www.jacksonimmuno.com/catalog/products/715-165-151).

Anti-IgG-mouse-647, Molecular Probes (#A31571), validated for immunofluorescence analysis, relevant citations can be found on the manufacturer's website (https://www.thermofisher.com/antibody/product/Donkey-anti-Mouse-IgG-H-L-Highly-Cross-Adsorbed-Secondary-Antibody-Polyclonal/A-31571).

Anti-AlexaFluor488 phalloidin, Molecular Probes (#A12379), validated for immunofluorescence analysis, relevant citations can be found on the manufacturer's website (https://www.thermofisher.com/order/catalog/product/fr/fr/A12379).

Anti-AlexaFluor546 phalloidin, Molecular Probes (#A22283), validated for immunofluorescence analysis, relevant citations can be

found on the manufacturer's website (https://www.thermofisher.com/order/catalog/product/fr/fr/A22283).

Anti-IgG-mouse-HRP, Jackson ImmunoResearch (#115-035-062), validated for Western Blot analysis, relevant citations can be found on the manufacturer's website (https://www.jacksonimmuno.com/catalog/products/115-035-062).

Anti-IgG-rabbit-HRP, Jackson ImmunoResearch (#111-035-045), validated for Western Blot analysis, relevant citations can be found on the manufacturer's website (https://www.jacksonimmuno.com/catalog/products/111-035-045).

Eukaryotic cell lines

Policy information about cell lines and Sex and Gender in Research

Cell line source(s)	Human MDA-MB-231 breast adenocarcinoma cells obtained from ATCC (ATCC HTB-26).<br>Human HT-1080 fibrosarcoma cells were obtained from ATCC (ATCC CCL-121).
Authentication	The two cell lines were not further authenticated.
Mycoplasma contamination	All cell lines used in this study are routinely tested for Mycoplasma contamination using a PCR-based approach and were negative for mycoplasma contamination.
Commonly misidentified lines<br>(See ICLAC register)	No commonly misidentified cell lines were used in this study.
