# Supplementary figures and images for: A mechanosensitive caveolae–invadosome interplay drives matrix remodelling for cancer cell invasion
Source: Nat Cell Biol. 2023 Oct 30;25(12):1787–803. doi: 10.1038/s41556-023-01272-z (PMC10709148; doi:10.1038/s41556-023-01272-z)

Extended Data Fig. 4c

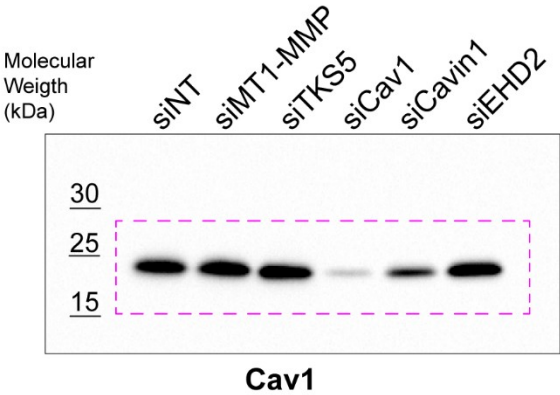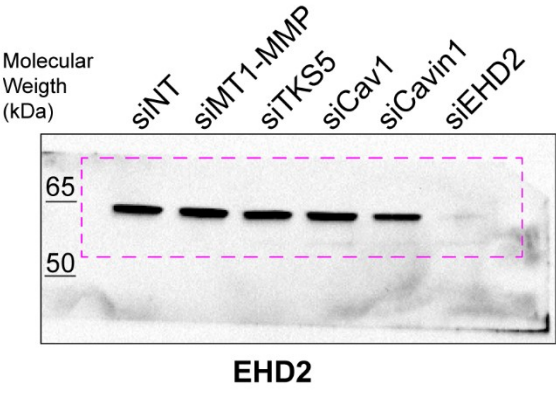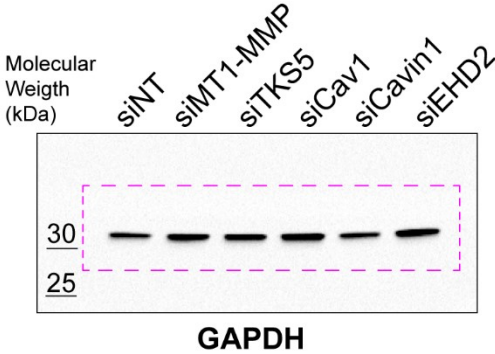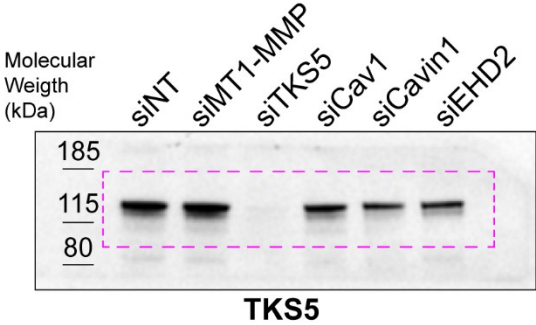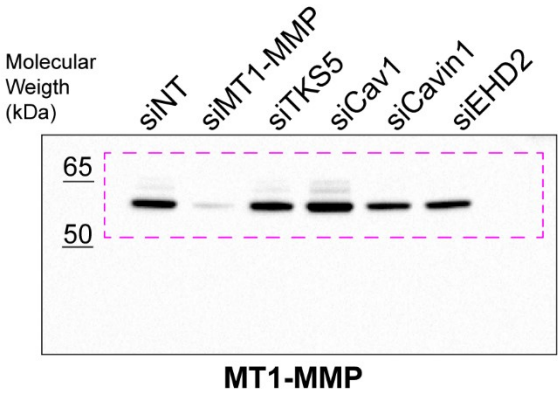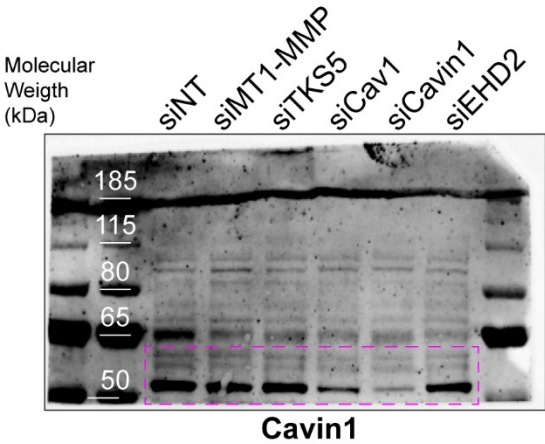

Supplement: Supplementary file 16 — Unprocessed western blots [file 41556_2023_1272_MOESM16_ESM.pdf]

Extended Data Fig. 5b

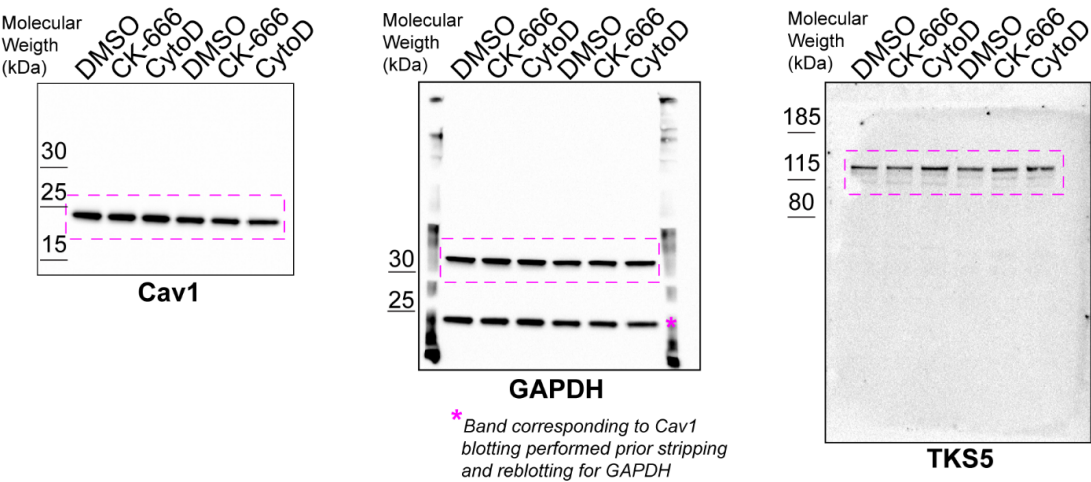

Extended Data Fig. 5c

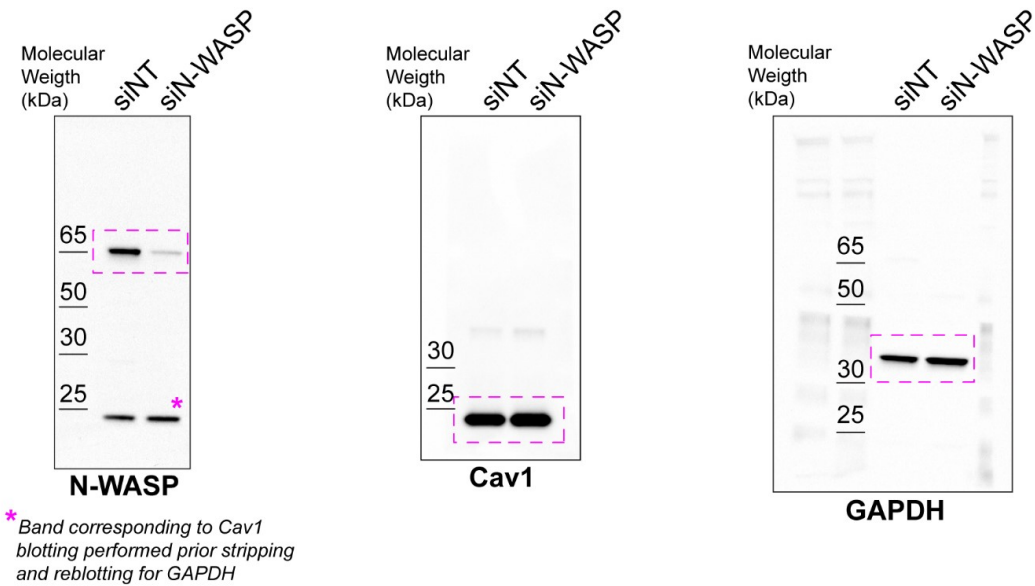

Extended Data Fig. 5m

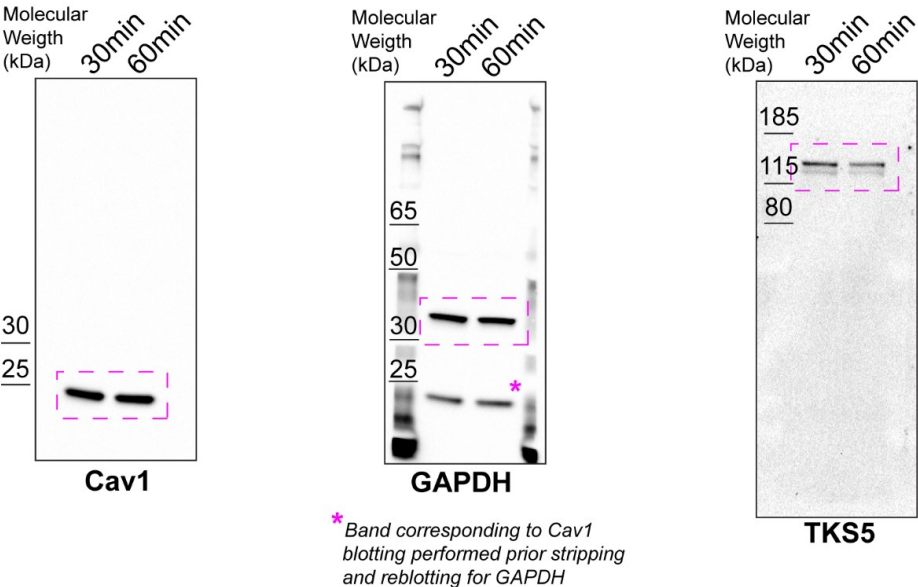

Supplement: Supplementary file 18 — Unprocessed western blots [file 41556_2023_1272_MOESM18_ESM.pdf]

Extended Data Fig. 6f

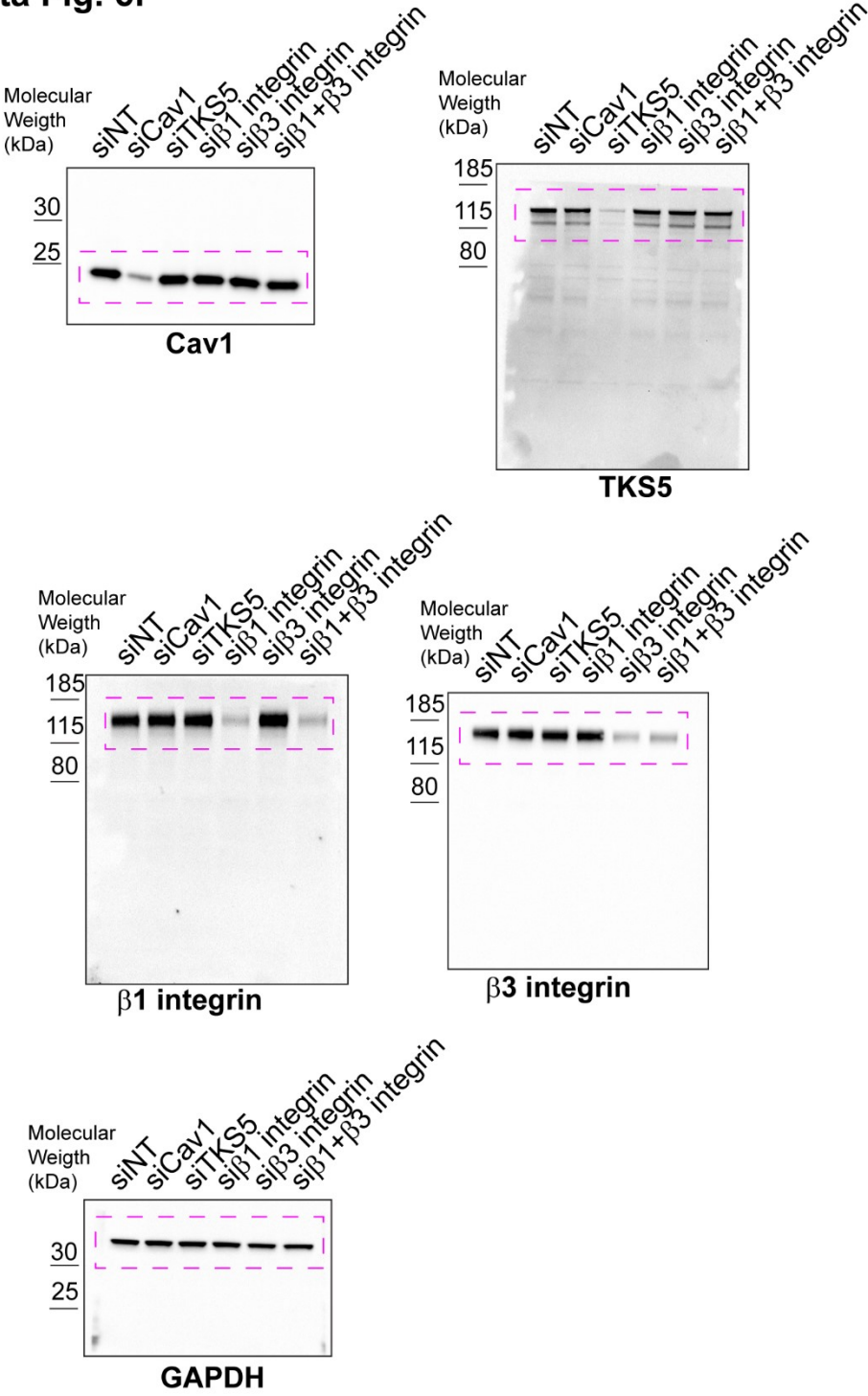

Supplement: Supplementary file 20 — Unprocessed western blots [file 41556_2023_1272_MOESM20_ESM.pdf]

Extended Data Fig. 9b

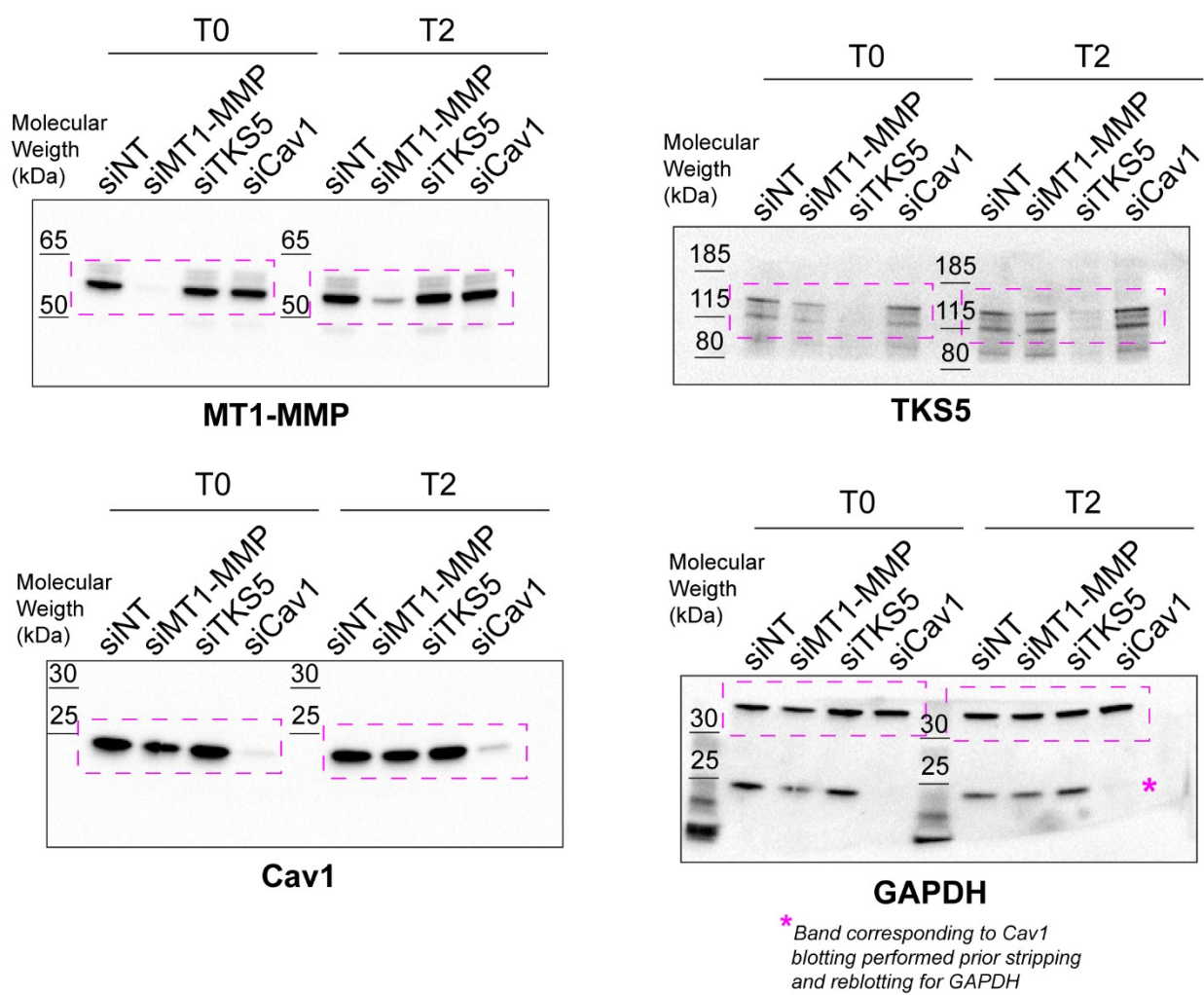

Supplement: Supplementary file 23 — Unprocessed western blots [file 41556_2023_1272_MOESM23_ESM.pdf]
